# Image-Based Robotic Unicompartmental Knee Arthroplasty Results in Fewer Radiologic Outliers with No Impact on Revision Rates Compared to Imageless Systems: A Systematic Review

**DOI:** 10.3390/jcm14175996

**Published:** 2025-08-25

**Authors:** Horia Tomescu, George M. Avram, Giacomo Pacchiarotti, Randa Elsheikh, Octav Russu, Andrej M. Nowakowski, Michael T. Hirschmann, Vlad Predescu

**Affiliations:** 1 Faculty of General Medicine, Carol Davila University of Medicine and Pharmacy, Bulevardul Eroii Sanitari Numarul 8, Sector 5, 050474 Bucuresti, Romania; horia.r.tomescu@stud.umfcd.ro; 2 Department of Orthopedic Surgery and Traumatology, Kantonsspital Baselland, 4101 Bruderholz, Switzerland; george-mihai.avram@ksbl.ch (G.M.A.); randa.el-sheikh@ksbl.ch (R.E.); andrej.nowakowski@ksbl.ch (A.M.N.); michael.hirschmann@unibas.ch (M.T.H.); 3 Department of Clinical Research, Research Group Michael T. Hirschmann, Regenerative Medicine & Biomechanics, University of Basel, CH-4001 Basel, Switzerland; 4 Department of Anatomy, Histology, Legal Medicine, and Orthopaedics, Sapienza University of Rome, Piazzale Aldo Moro, 5, 00185 Roma, Italy; giacomo.pacchiarotti@uniroma1.it; 5 Faculty of General Medicine, George Emil Palade University of Medicine, Pharmacy, Science, and Technology of Târgu Mureș, 540139 Targu Mures, Romania; 6 Orthopaedic and Traumatology Department, George Emil Palade University of Medicine, Pharmacy, Science, and Technology of Târgu Mureș, 540139 Targu Mures, Romania; 7 George Emil Palade University of Medicine, Pharmacy, Science, and Technology of Târgu Mureș, 38 Gh. Marinescu Street, 540142 Târgu Mureș, Romania; 8 Orthopaedics and Traumatology Department, Ponderas Academic Hospital, 014142 Bucharest, Romania; vlad.predescu@reginamaria.ro

**Keywords:** robotic-assisted, unicompartmental knee arthroplasty, imageless, image-based, revisions, alignment, Mako, Navio

## Abstract

**Background**: Robotic-assisted unicompartmental knee arthroplasty (UKA) enhances the precision of component alignment compared to conventional techniques. Although various robotic systems exist, direct comparisons assessing their relative clinical performance remain limited. The purpose of this study is to provide a comparison between image-based and imageless robotic UKA. **Methods**: A systematic review was conducted in accordance with PRISMA guidelines. Five databases were searched: PubMed (via MEDLINE), Epistemonikos, Cochrane Library, Web of Science, and Scopus. Inclusion criteria were (1) studies comparing rUKA and cUKA with radiologic parameters and revision rates (prospective or retrospective), (2) human subjects, (3) meta-analyses for cross-referencing, and (4) English language. Data collected included (1) pre- and postoperative radiologic parameters, (2) radiologic outliers, and (3) revisions and their causes. A random-effects meta-analysis was employed to enable a generalizable comparison. Mean differences (MDs) with 95% confidence intervals (CIs) were calculated for continuous variables, and log odds ratios (LORs) with 95% CIs for binary outcomes. **Results**: Image-based robotic UKA was associated with fewer joint line height outliers (LOR = 3.5, 95% CI: 0.69–6.30, *p* = 0.015) using a 2° threshold. HKA outliers (thresholds 2–3°) were also reduced (LOR = 0.6, 95% CI: 0.09–1.19, *p* = 0.024). Posterior tibial and posterior femoral implant fit were significantly lower with image-based systems (LOR = 1.7, 95% CI: 1.37–2.03, respectively, LOR = 1.7, 95% CI: 1.29–1.91; *p* < 0.001 for both). No significant differences in revision rates were observed. **Conclusions**: Image-based robotic systems may result in fewer outliers in key radiologic parameters, including hip–knee angle, joint-line height, posterior tibial, and posterior femoral fit, though reporting remains highly heterogeneous.

## 1. Introduction

Unicompartmental knee arthroplasty (UKA) has been historically shown to have higher revision rates than total knee arthroplasty (TKA) [1,2]. Causes for UKA failure include progression of osteoarthritis in other compartments and technical factors such as component malpositioning, that leads to knee anatomy changes [3,4,5,6]. To address the critical importance of implant alignment and soft-tissue balance in UKA, robotic-assisted surgical systems have been introduced with the aim of enhancing the precision and reproducibility of implant placement [7,8]. Several distinct robotic systems are currently utilized in UKA, from imageless to image-based, from closed systems dedicated to specific implant models to open systems allowing more implant choice [9]. While image-based robotic systems rely on detailed preoperative imaging (CT or MRI) to construct an accurate 3D anatomical model for surgical planning, imageless robotic systems, on the other hand, forego any pre-op CT/MRI. They involve intraoperative data acquisition through mapping of the patient’s bony landmarks and joint surfaces. In practice, both approaches aim to improve implant positioning relative to manual techniques, but their workflows differ in planning and execution [10,11].

Robotic assistance, in general, has been shown to markedly improve the precision of component alignment, yielding far fewer outliers compared to conventional UKA instrumentation [12]. The superiority of the robotic technology was also highlighted in studies examining revision rates, robotics significantly reducing them at mid- to long term follow up [13]. When image-based versus imageless robots are compared, evidence suggests that image-based systems may confer an implantation accuracy advantage compared to imageless systems [14]. Still, both types of robotic assistance greatly enhance surgical accuracy and consistency relative to conventional instrumentation [15], which is evidenced by significantly reduced alignment variability and instruments alignment errors in robotic-assisted UKA [16,17]. The net effect should be an improvement in implant longevity and knee function. Nonetheless, these gains in alignment precision and implant fit remain an area of ongoing research as their direct link to patient-reported outcome measures remains unclear, given that anatomy restoration is not clearly described in relation to knee kinematics [18]. These findings highlight that factors beyond radiographic alignment, such as patient selection, soft-tissue management, and postoperative rehabilitation, also drive patient outcomes [19,20,21,22]. Surgical accuracy alone may not immediately translate to a noticeable clinical benefit in every case, especially in the early postoperative period [23,24].

No single robotic system has demonstrated clearly superior clinical outcomes, and comparative studies are limited. Therefore, a comprehensive comparison between robotic systems would provide surgeons with the needed evidence to accompany their decision on which robotic system to use in their practice. The purpose of this study is to make an indirect comparison between two of the commonly used robotic systems in UKA.

## 2. Materials and Methods

### 2.1. Information Sources and Eligibility Criteria

This systematic review was conducted in accordance with PRISMA (Preferred Reporting Items for Systematic Reviews and Meta-Analyses) guidelines and was prospectively registered with PROSPERO (CRD42024595082). This analysis represents a secondary review of data previously collected for a systematic review that compares robotic and conventional unicompartmental knee arthroplasty. A comprehensive literature search was performed on 24 March 2024, from inception, across five electronic databases: MEDLINE (via PubMed), Epistemonikos, Cochrane Library, Web of Science, and Scopus. The full search strategy is provided in Appendix A.

The inclusion criteria were as follows: (1) studies comparing rUKA and cUKA with reported radiologic parameters and revision rates (prospective or retrospective designs), (2) human studies, (3) meta-analyses for cross-referencing, and (4) English language. This analysis focused exclusively on studies involving the MAKO (image-based) and Navio (imageless) robotic systems, as they represented the majority of robotic platforms identified in the included literature. Studies reporting on lateral UKA, navigation results, cadaveric studies, editorials, commentaries, surgical techniques, letters to the editor, or study protocols were excluded.

### 2.2. Data Extraction and Analysis

**Figure 1:**
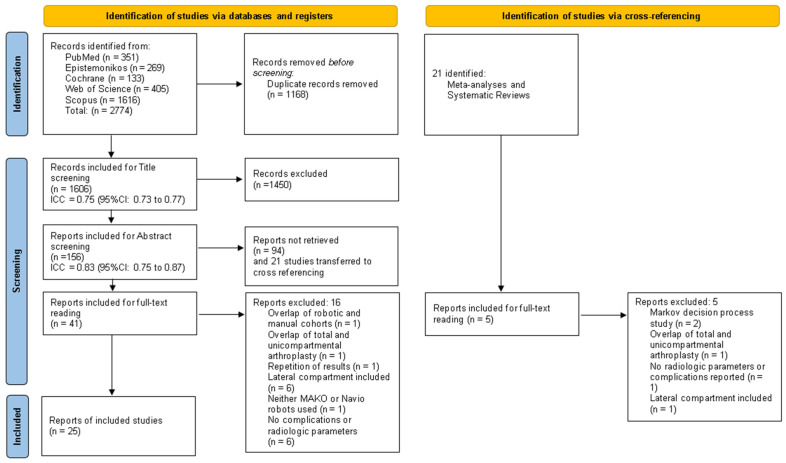
PRISMA flow diagram.

Studies identified from databases, following duplicate removal, were included in the selection process. Due to the limited number of studies directly comparing robotic systems, robotic cohorts were extracted from studies comparing robotic-assisted and conventional techniques. The selection process was conducted by four reviewers and consisted of three stages: title screening, abstract screening, and full-text screening. During the title screening phase, the records were evenly divided, each half being assigned to a group of two reviewers. Inter-rater agreement was calculated and reported as the intraclass correlation coefficient (ICC) with a 95% confidence interval, as shown in Figure 1, for both title and abstract screening. Discrepancies were resolved through discussion among all four reviewers.

During the cross-referencing phase all identified systematic reviews and meta-analyses were equally distributed among three of the reviewers, who extracted all studies meeting the inclusion and exclusion criteria. These were then compared against the studies identified in the initial literature search.

A total of 2774 studies were identified, and after duplicate removal, 1606 papers were left for title screening. This was further narrowed down to 156 studies for abstract screening. The inter-rater agreement was 0.75 (95% CI: 0.73 to 0.77) for title screening and 0.83 (95% CI: 0.75 to 0.87) for abstract screening. 21 meta-analyses and systematic reviews were identified. From cross-referencing, no additional studies met the inclusion and exclusion criteria. Ultimately, 41 studies were included for full-text screening, of which 16 were excluded, leaving 25 studies for final data extraction (Figure 1).

### 2.3. Study Quality, Bias, and Characteristics

The Newcastle-Ottawa Scale (NOS) was used to assess risk of bias in the included studies, and the Oxford Centre for Evidence-Based Medicine (OCEBM) classification was used to assess the levels of evidence. Approximately half of the studies included in the analysis were classified as low quality, with NOS scores below 7. The remaining studies were deemed high quality, with NOS scores ranging from 7 to 9. Concerns regarding potential biases were identified, particularly in the selection of study participants, which may compromise the comparability of study cohorts. Additionally, there were notable risks of bias in outcome measurement, primarily due to inadequate follow-up protocols. A detailed analysis of risk of bias (Table S1) and level of evidence (Table S2) is available in the Supplementary files.

### 2.4. Data Collection Process and Data Items

The studies included for full-text screening were split in half, and two reviewers were assigned for data collection for each half. The following general data were collected: (1) authors, (2) level of evidence, (3) robot type, (4) demographics—patient age, gender, BMI, follow-up, and (5) operative time. Clinically relevant data points were as follows: (1) pre- and postoperative radiologic parameters, (2) radiologic outliers, and (3) revisions and reasons for revision. Only studies reporting on both preoperative and postoperative HKA were analyzed.

### 2.5. Statistical Analysis and Data Synthesis

The data collected included mean, standard deviation (SD), median, min, max, interquartile ranges, and count, as appropriate. Weighted means and SDs were calculated. A random-effects meta-analysis model was employed to enable a generalizable comparison while accounting for between-study heterogeneity. For continuous variables, mean differences (MDs) with 95% confidence intervals (CIs) were calculated. For binary outcomes, log odds ratios (LORs) with 95% CIs were used. Results are presented in tables. Positive MD and LOR indicate higher values for the imageless group. The transformation method proposed by Wan et al. was used to estimate means and SDs when only medians and ranges or interquartile ranges were reported [25]. SPSS (IBM SPSS Statistics 29.0.0.0) was used for statistical analysis.

Revision analysis was performed on four main categories: (1) total number of revisions, (2) revisions to total knee arthroplasty (TKA), (3) revisions for persistent pain, and (4) revisions for aseptic loosening.

## 3. Results

### 3.1. Patient Demographics

A total of 1622 patients were included in the radiological analysis. Relevant patient demographics such as age, gender, BMI, and operative time are reported in Table 1.

### 3.2. Planning Methodology and Measurements

Planning methodology was not always presented, only 2/3 image-based system studies reporting HKA targets [28,32], while 0/2 imageless system studies reported it. Out of the 6 image-based studies reporting postoperative tibial slope values, 4 presented their target values [16,28,30,32], while 2/4 did the same for the imageless group [17,41].

The femoral sagittal angle was assessed either as the angle between a line along the anterior cortex of the metaphyseal-diaphyseal junction and a line through the posterior peg of the femoral component [26,41], or as the angle between the femoral axis and the femoral peg [27]. The values for the femoral sagittal angle from latter study [27] were excluded from the analysis due to a distinct orientation of the prosthesis compared to the ones used in the other studies [26,41]. The tibial coronal angle, or the tibial component coronal alignment, was measured as the angle between the tibial mechanical axis and the medial to lateral axis of the tibial implant [16,27,28,29,30,42]. Three types of congruencies were described between the tibial component and the tibia: medial, anterior and posterior. All three were measured as the distance between the tip of the tibial component and the edge of the tibial plateau, either medially [8,31], anteriorly [8,31] or posteriorly [8,27,31]. The posterior femur fit was defined as the distance from the posterior tip of the femoral component to the posterior part of the femoral condyle [8,27].

### 3.3. Preoperative Radiologic Measurements

Preoperative radiologic measurements from included studies are summarized in Table 2. Three studies reported both pre- and postoperative HKA for the image-based group, and two for the imageless group. Only one study per group reported tibial slope.

### 3.4. Postoperative Radiologic Measurements

Postoperative radiologic measurements are presented in Table 2. Although both pre- and postoperative HKA were significantly different between the two groups (*p* = 0.044 and *p* < 0.001), preoperative MD was −0.7 (95% CI: −1.38 to −0.02), which increased to a postoperative MD of −2.1 (95% CI: −2.62 to −1.58). Similarly, preoperative tibial slope saw an MD reduction of 1.4, from 3.1 (95% CI: 2.18 to 4.03) (*p* < 0.001) to 1.7 (95% CI: 1.37 to 2.03).

There were also statistically significant differences (*p* < 0.001) between postoperative values for all the implant components’ alignment measurements (Table 3). While MDs for most of the aforementioned parameters remained at a maximum of 2.5, the MD for the femoral sagittal angle was 10.2 (95% CI: 7.66 to 12.74).

### 3.5. Outliers

There were different thresholds for HKA outliers (Table 4). For studies on image-based systems, they were 2° [32,33] and 3° [28], while for imageless systems they were 2° [17] and outside of 175–180° [42]. For joint line height, all studies had the same threshold of 2°, with LOR of 3.5 (95% CI: 0.69 to 6.30) with *p* = 0.015.

Out of the four implant position angles, the femoral sagittal angle was the only one for which outliers were recorded (Table 4). The thresholds were as follows: 2° [34] and 15° of flexion [31] for image-based studies, and 3° with a target of 45° [41] for the imageless system.

All four congruencies had outlier thresholds of 3 mm [8] in the imageless group. Image-based studies had thresholds of 3 mm for posterior femur fit [8] and anterior tibia fit [8,31], and both 2 mm [31] and 3 mm [8] for medial tibia fit and posterior tibia fit (Table 4).

### 3.6. Revision Rate Analysis

Sixteen studies were included for revision analysis as depicted in Table 5. A total of 583 patients operated on with the image-based system and 486 with the imageless system were analyzed. There were no statistically significant differences in total revisions at either all follow-ups or at a minimum of 12 months.

## 4. Discussion

The findings indicate that robotic assistance type in unicompartmental knee arthroplasty might influence perioperative and radiographic outcomes, although both systems ultimately achieved similar clinical results as depicted by the revision rates. The most striking differences were observed in postoperative limb alignment and the frequency of alignment outliers, especially joint line height and posterior tibial fit, which are relevant to implant performance [33]. Image-based assisted UKA resulted in a postoperative alignment (HKA) closer to neutral on average (mean ~178.7°) compared to the imageless group (~176.6°). Importantly, HKA is a confounded variable influenced by the preoperative anatomy and surgical balancing and should not be interpreted in isolation. This 2° difference, while statistically significant, should be interpreted within the context of each robotic system and patient-specific alignment. Notably, alignment outliers beyond target ranges were less common with the image-based system—only about 17.5% of image-based robotic cases fell outside alignment goals for HKA versus 28.7% of imageless cases. In other words, the image-based cohort had fewer alignment deviations from intended planning, suggesting more consistency in restoring the limb axis. However, one should be aware that the outlier concept is highly heterogeneous [28,32]. While some studies consider outliers outside of 3° HKA, others might accept up to 5° [31] or seek to reproduce native anatomy without boundaries. This is consistent with prior evidence that image-based robotic systems confer a precision advantage, yielding tighter clustering of implant alignment [14]. However, the mean alignment achieved with imageless systems (approximately 3° of varus from neutral) may actually reflect an intentional slight under-correction within the patient’s native laxity [4,47]. The imageless group’s alignment outcomes align with this philosophy, whereas the image-based group’s alignment was closer to the mechanical neutral.

Crucially, despite the differences in radiographic alignment, revision rates did not significantly differ between image-based and imageless systems in the observed follow-up period. The overall revision rate was low in both groups and statistically equivalent, but lower compared to manually implanted UKA, especially when longer follow-up points are considered [13]. Even when excluding studies with follow-ups of less than 12 months, the revision frequencies remained comparable (2.7% vs. 3.3%). This suggests that neither robotic system has translated its technical differences into a detectable disparity in implant survival. In particular, the rate of conversion to total knee arthroplasty TKA, arguably the most clinically significant failure mode for UKA seems to favor the image-based group (0% vs. 2–3% in the imageless group at ≥1 year), but the difference did not reach statistical significance. No significant differences were observed in revisions performed for specific reasons, such as aseptic loosening or persistent pain, between the two systems, although the low rates of revision are consistent with existent literature [48]. These findings echo the results of other early outcome studies that report no divergence in clinical success between image-based and imageless systems. For example, there was no difference in 1-year functional scores or alignment between the two systems in a prospective cohort [49]. It was also noted that at 2-year follow-up, both MAKO and Navio yielded equally favorable clinical outcomes (KOOS, pain relief, ROM) with no significant inter-system differences [50]. The present analysis reinforces this parity in short-term clinical performance: despite some variations in how each system achieves component placement, both robotic platforms appear to confer low early failure rates. This underscores an important point, radiographic perfection does not necessarily equate to short- or mid-term clinical superiority. The current study also revealed significant differences in implant positioning within the proximal tibia and both systems’ ability to reconstruct the proximal tibia osseous fit. For instance, the mean tibial component coronal angle (deviation of the tibial implant from 90°) was lower in the image-based group (1.4°) than in the imageless group (3.9°), implying the Mako implants were either set with less varus/valgus error on average or intentionally implanted more neutral. Furthermore, the influence of patient anatomy and surgical decisions could not be investigated given that current literature focuses on reporting mean values instead of patient-specific alignment goals. Similarly, the femoral component’s sagittal orientation differed by about 10° between groups: image-based femoral components were implanted in a mean flexion of ~37.6°, whereas imageless-assisted cases averaged ~47.8° flexion. This large angular discrepancy likely stems from differing surgical workflows and implant designs [51,52,53,54] as the literature has reported, there is still room for improvement in overall implant–bone coverage [55]. Measures of implant fit also favored the image-based system in our analysis. Posterior tibial fit was, on average, more optimal in the image-based group (mean 3.4 mm mismatch) compared to the imageless group (5.1 mm). On the other hand, although still significant, one study comparing imageless with image-based robotics found a difference of only 0.6 mm mismatch [8]. A similar trend was seen for posterior femoral fit (3.5 mm vs. 5.1 mm) [8]. These differences suggest that Mako’s image-guided planning allowed surgeons to achieve a closer match to the intended implant position, potentially minimizing overhang or uneven coverage at the edges of the components. In practical terms, better component fit might reduce edge-loading and wear, but whether a 1–2 mm average difference in fit yields any functional benefit is not established [56,57]. When we examined outlier rates, we found that imageless robotic UKAs had a significantly higher incidence of outliers in posterior tibial fit. Over 31% of imageless cases were categorized as outliers in posterior tibial fit, compared to only 4.5% of image-based cases. This implies that a considerable subset of imageless cases had notably suboptimal tibial component seating, whereas image-based cases were consistently within acceptable bounds. Nonetheless, this aspect should be interpreted with caution due to the fact that multiple implant types were used with the imageless platform while for the image-based platform only one (Table S2, Supplemental File). It should be emphasized, however, that for other fit parameters, the systems performed similarly, suggesting a probable sizing issue. Overall, both robotic platforms dramatically limited the occurrence of gross outliers across all measured parameters when compared to historical reports of conventional UKA, which supports the general advantage of robotic assistance in achieving reliable implantation [58,59].

Finally, this study has several limitations. Due to the limited literature on other robotic systems, the decision to include only studies focused on Mako and Navio limited our study’s capacity to provide robust evidence on the different robotic technologies available. This review is further limited by the lack of data on planned versus executed implant position, forcing the authors to focus on outliers of broader radiographic variables that may have little impact on UKA outcomes, while PROMS and revision rates may also be influenced by geometric differences between implants used in the two systems. Furthermore, this is an indirect comparison, as the results are based on studies comparing robotic with conventional implantation, rather than directly comparing image-based and imageless robotic techniques. This is because the current literature offers very limited evidence on differences between these two robotic approaches in UKA implantation. Another important limitation is that alignment angles, outliers, and fit measurements are highly heterogeneous measures that are mostly influenced by the surgical team’s workflows, training, and preferences. For this reason, it is highly probable that the reported results are confounded by these factors, leaving PROMs and revision rates as the best available methods of assessing the performance of each robotic system. Nonetheless, future efforts should strive to provide a common analysis framework, as without it, concrete data on implantation accuracy cannot be transparently assessed, leaving a large room for interpretation and bias. Lastly, to enable robust analyses, more consistent reporting of outcomes after robotic UKA is needed, as this study highlights the challenges of conducting meaningful subgroup analyses.

## 5. Conclusions

Current evidence on fixed-bearing UKA implantation performance of image-based and imageless robotic systems cannot provide undeniable evidence in support of a single robotic platform, as both show comparable implant survival at short- and mid-term follow-up, especially when contrasted with available evidence on conventional implantation. Implant design, which affects fit parameters, and implantation strategies are relevant confounders, together with the employed alignment targets. Studies should adopt more transparent methodologies and standardize the reporting of the aforementioned parameters.

## Figures and Tables

**Table 1:** Demographics of both Image-based and Imageless groups.

	Image-Based	Imageless	*p*-Value
Total Included	970 [8,16,23,26,27,28,29,30,31,32,33,34,35,36,37,38,39]	652 [8,17,40,41,42,43,44,45,46]	-
Age * (years)	64.2 (8.7) [8,23,26,27,28,29,31,32,33,34,35,36,37,38]	64.2 (9) [8,17,41,42,43,44,45,46]	1
Gender (M/F) (%)	42.8/57.2 [8,23,26,27,28,29,32,33,35,36,37,38]	47.6/52.4 [8,41,42,43,44,45,46]	-
Operative Time * (minutes)	90.7 (18) [16,26,29,30]	80.7 (15.2) [41,42,45]	<0.001
BMI *	28.4 (4.4) [8,23,28,29,31,33,35,36]	27.8 (4) [8,17,42,44,45,46]	0.023

* Data presented as Mean (SD).

**Table 2:** Preoperative and postoperative reported HKA, and tibial slope.

	Image-Based	Imageless	Mean Difference (95% CI)	*p*-Value
Preoperative HKA *	174.6 (3.3) [28,32,33]	173.9 (2.4) [42,44]	−0.7 (−1.38; −0.02)	0.044
Preoperative Tibial Slope *	78.7 (3.2) [26]	81.8 (2.3) [42]	3.1 (2.18; 4.03)	<0.001
Postoperative HKA *	178.7 (2.4) [28,32,33]	176.6 (2.2) [42,44]	−2.1 (−2.62; −1.58)	<0.001
Postoperative Tibial Slope *	83.9 (2.3) [16,26,28,29,30,32]	85.6 (1.9) [17,41,42,44]	1.7 (1.37; 2.03)	<0.001

* Data presented as Mean (SD).

**Table 3:** Postoperative mean values of femoral sagittal angle, tibial coronal angle, posterior tibia, and posterior femur fit.

	Image-Based	Imageless	Mean Difference (95% CI)	*p*-Value
Femoral Sagittal Angle *	37.6 (5.4) [26]	47.8 (4.2) [41]	10.2 (7.66; 12.74)	<0.001
Tibial Coronal Angle *	1.4 (2.7) [16,27,28,29,30]	3.9 (2.5) [42]	2.5 (2.02; 2.97)	<0.001
Fit Posterior Tibia (mm) *	3.4 (1.4) [8,27]	5.1 (1.1) [8]	1.7 (1.37; 2.03)	<0.001
Fit Posterior Femur (mm) *	3.4 (1.1) [8,27]	5.1 (1.2) [8]	1.7 (1.29; 1.91)	<0.001

* Data presented as Mean (SD).

**Table 4:** Outliers for: HKA, joint-line height, femoral sagittal angle, medial, anterior, posterior tibia, and posterior femur fit.

	Image-Based	Imageless	Log Odds Ratio (95% CI)	*p*-Value
Outliers HKA (%)	17.5 [28,32,33]	28.7 [17,42]	0.6 (0.09; 1.19)	0.024
Outliers Joint Line Height (%)	0 [33]	31.6 [42,43]	3.5 (0.69; 6.30)	0.015
Outliers Femoral Sagittal Angle (%)	27.8 [31,34]	25 [41]	−0.1 (−1.33; 1.05)	0.814
Outliers Fit Medial Tibia (%)	3.4 [8,31]	0 [8]	−1.9 (−4.83; 0.94)	0.186
Outliers Fit Anterior Tibia (%)	2.8 [8,31]	1.1 [8]	−1 (−3.13; 1.19)	0.378
Outliers Fit Posterior Tibia (%)	4.5 [8,31]	31.2 [8]	2.3 (1.44; 3.10)	<0.001
Outliers Fit Posterior Femur (%)	12.9 [8]	7.5 [8]	−0.6 (−1.58; 0.38)	0.231

**Table 5:** Revision rates reported at a minimum follow-up of 1.5 months or 12 months.

	Image-Based	Imageless	Log Odds Ratio (95% CI)	*p*-Value
All Follow-up Periods				
Total Included	583 [16,23,26,29,30,35,36,37,38,39]	486 [17,41,42,44,45,46]	-	-
Mean Follow-up (months)	23.7	30.5	-	-
Total Revisions (%)	2.23	2.88	0.26 (−0.50; 1.03)	0.501
Revisions to TKA (%)	0	2.06	2.78 (−0.06; 5.62)	0.055
Revisions for Pain (%)	0.68	0.41	−0.51 (−2.22; 1.19)	0.554
Revisions for Aseptic Loosening (%)	1.03	1.65	0.476 (−0.59; 1.54)	0.381
Minimum 12 Months Follow-up				
Total Included	479 [16,23,26,29,30,37,38,39]	304 [17,42,46]	-	-
Mean Follow-up (months)	28.2	46.5	-	-
Total Revisions (%)	2.71	3.29	0.20 (−0.64; 1.04)	0.643
Revisions to TKA (%)	0	2.96	2.82 (−0.03; 5.67)	0.052
Revisions for Pain (%)	0.63	0.33	−0.65 (−2.92; 1.62)	0.576
Revisions for Aseptic Loosening (%)	1.25	2.63	0.76 (−0.31; 1.83)	0.165

## Data Availability

All data are available on reasonable request.

## Supplementary Materials

The following supporting information can be downloaded at: https://www.mdpi.com/article/10.3390/jcm14175996/s1, Table S1: Risk of bias assessment; Table S2: Levels of evidence and study characteristics.

## Appendix A

Search string: PubMed: Robot* AND (Unic* OR Partial OR UKA) AND Knee; Web of Science: ((ALL = (Robot*) AND (ALL = (Unic*) OR ALL = (UKA) OR ALL = (Partial))) AND ALL = (Knee)); Scopus: ALL (knee) AND ALL (robot*) AND ALL (unic*) AND (LIMIT-TO (LANGUAGE, “English”)) AND (LIMIT-TO (EXACTKEYWORD, “Human”) OR LIMIT-TO (EXACTKEYWORD, “Humans”) OR LIMIT-TO (EXACTKEY WORD, “Knee”) ORLIMIT-TO (EXACTKEYWORD, “Arthroplasty, Replacement, Knee”)); Epistemonikos: knee AND robot* AND uni* OR partial; Cochrane: knee AND robot* AND uni* OR partial.
