# Supplementary material for: Image-Based Robotic Unicompartmental Knee Arthroplasty Results in Fewer Radiologic Outliers with No Impact on Revision Rates Compared to Imageless Systems: A Systematic Review
Source: J Clin Med. 2025 Aug 25;14(17):5996. doi: 10.3390/jcm14175996 (PMC12428876; doi:10.3390/jcm14175996)
Supplement: Supplementary file 1 [file jcm-14-05996-s001.zip › jcm-3757584-supplementary.pdf]

Table S1. Risk of bias assessment.

Study	Selection (4 max)	Comparability (2 max)	Outcome (3 max)	Total (9 max)
MacCallum et al. (2016)	★★	0	★★★	★★★★★
Wu et al. (2021)	★★★★	★	★★	★★★★★★★
Yeung et al. (2023)	★★★★	★	★★	★★★★★★★
Hansen et al. (2014)	★★★	0	★★	★★★★★
Wong et al. (2019)	★★★	★	★★	★★★★★★
Kwon et al. (2024)	★★★★	★★	★★	★★★★★★★★
Kayani et al. (2019)	★★★★	★	★★	★★★★★★★
Kazarian et al. (2021)	★★★★	★	★	★★★★★★
Park et al. (2019)	★★★★	0	★★	★★★★★★
Gilmour et al. (2018)	★★★★	★★	★★★	★★★★★★★★★
Clement et al. (2023)	★★★★	★★	★★★	★★★★★★★★★
Thilak et al. (2020)	★★★★	★	★	★★★★★★
Çabuk et al. (2022)	★★★★	★	★	★★★★★★
Banger et al. (2021)	★★★★	★★	★★★	★★★★★★★★★
Bell et al. (2016)	★★★★	★★	★★	★★★★★★★★
Lonner et al. (2010)	★★★★	0	★	★★★★★
Batailler et al. (2021)	★★★★	★★	★★	★★★★★★★★
Batailler et al. (2019)	★★★★	★★	★★★	★★★★★★★★★
Batailler et al. (2023)	★★★★	★★	★★	★★★★★★★★
Negrin et al. (2021)	★★★★	★	★	★★★★★★
Goh et al. (2022)	★★★★	0	★★	★★★★★★
Foissey et al. (2023)	★★★★	★	★★★	★★★★★★★★
Crizer et al. (2021)	★★★	0	★★	★★★★★
Herry et al. (2017)	★★★★	★★	★★	★★★★★★★★
Kumar et al. (2024)	★★★★	★	★	★★★★★★

Table S2. Levels of evidence and study characteristics.

Study	Robot	Sample size	Implant	Study Type	Evidence Level
MacCallum et al. (2016)	MAKO	87	Restoris	Prospective comparative	III
Wu et al. (2021)	MAKO	52	Restoris	Retrospective cohort	III
Yeung et al. (2023)	MAKO	74	Restoris	Case-Control	IV
Hansen et al. (2014)	MAKO	30	Restoris	Retrospective cohort	III
Wong et al. (2019)	MAKO	58	Restoris	Retrospective cohort	III
Kwon et al. (2024)	MAKO	35	Restoris	Retrospective cohort	III
Kayani et al. (2019)	MAKO	73	Restoris	Prospective comparative	III
Kazarian et al. (2021)	MAKO	86	Restoris	Retrospective cohort	III
Park et al. (2019)	MAKO	55	Restoris	Retrospective cohort	III
Gilmour et al. (2018)	MAKO	58	Restoris	RCT	II
Clement et al. (2023)	MAKO	65	Restoris	RCT	II
Thilak et al. (2020)	MAKO	24	Restoris	Case Series	IV
Çabuk et al. (2022)	MAKO	36	Restoris	RCT	II
Banger et al. (2021)	MAKO	55	Restoris	RCT	II
Bell et al. (2016)	MAKO	58	Restoris	RCT	II
Lonner et al. (2010)	MAKO	31	Restoris	Prospective comparative	III
Batailler et al. (2021)	MAKO & Navio	93 & 93	Restoris & Journey	Retrospective cohort	III
Batailler et al. (2019)	Navio	57	HLS Uni Evolution	Case-Control	IV
Batailler et al. (2023)	Navio	33	Journey	RCT	II
Negrin et al. (2021)	Navio	16	Journey	Retrospective cohort	III
Goh et al. (2022)	Navio	133	STRIDE	Cost analysis	IV
Foissey et al. (2023)	Navio	197	Journey	RCT	II
Crizer et al. (2021)	Navio	50	STRIDE	Retrospective cohort	III
Herry et al. (2017)	Navio	23	HLS Uni Evolution	Case-Control	IV
Kumar et al. (2024)	Navio	50	Journey	Retrospective cohort	III
